# Influence of psychosocial and health-seeking behaviour on the risk of falling among persons living with type 2 diabetes in the Malaysian Elders Longitudinal Research (MELoR) cohort

**DOI:** 10.1007/s40520-025-02961-5

**Published:** 2025-03-11

**Authors:** Sheron Sir Loon Goh, Foong-Ming Moy, Sumaiyah Mat, Shazeea Mohamed Ali, Zi Xin Hoo, Sai Ganesh Rao Apparoo, Maw Pin Tan

**Affiliations:** 1 Department of Clinical Pharmacy and Pharmacy Practice, Faculty of Pharmacy, Universiti Malaya, Kuala Lumpur, Malaysia; 2 Centre of Epidemiology and Evidence Based Practice, Department of Social and Preventive Medicine, Faculty of Medicine, Universiti Malaya, Kuala Lumpur, Malaysia; 3 Ageing and Age-Associated Disorders Research Group, Faculty of Medicine, Universiti Malaya, Kuala Lumpur, Malaysia; 4 Centre for Healthy Aging and Wellness, Faculty of Health Sciences, Universiti Kebangsaan Malaysia, Kuala Lumpur, Malaysia; 5 Department of Geriatric Medicine, Austin Hospital, Melbourne, Australia; 6 Faculty of Medicine, Universiti Malaya, Kuala Lumpur, Malaysia; 7 Division of Geriatric Medicine, Department of Medicine, Department of Primary Care Medicine, Faculty of Medicine, Universiti Malaya, 50603 Kuala Lumpur, Malaysia

**Keywords:** Diabetes, Falls, Psychosocial factors and older persons

## Abstract

**Background:**

Older persons with diabetes have an increased falls risk that could lead to serious complications including death.

**Aim:**

To determine the influence of psychosocial factors and health-seeking behaviour on the risk of falling among individuals with type 2 diabetes.

**Methods:**

This prospective study included community-dwelling adults aged ≥55 years selected through stratified random sampling from three neighbouring parliamentary constituencies. Data was collected at baseline in 2013–2015 with computer-assisted home-based interviews and follow-up in 2019 via telephone interviews.

**Results:**

Data on diabetes status and falls were available for 908 participants at baseline and follow-up. Diabetes was present in 42.2% of included participants at follow-up, of whom 22.8% at baseline and 25.3% at 5-year follow-up had at least one fall within the last 12 months. Diabetics had a higher risk of falls at baseline (OR: 1.484; 95% CI: 1.060–2.077) and follow-up (OR: 1.424; 95% Cl: 1.038–1.954) than non-diabetics. It was found that female gender, arthritis, alcohol and presence of depression anxiety or stress were associated with increased risk of falls in diabetics. The presence of any depression, anxiety or stress remained significantly associated with falls in diabetics (OR: 1.947; 95% Cl: 1.115–3.402) after adjustments for age, gender, ethnicity, and education but this relationship was attenuated after additional adjustment for arthritis (OR: 1.763; 95% CI: 0.996–3.122).

**Conclusion:**

Our findings suggest that psychological issues are significantly associated with increased risk of falls at five-year follow-up in individuals aged 55 years and over with diabetes. These findings highlight the potential importance of psychosocial support among diabetics to reduce the risk of falls, improve patient outcomes and quality of life.

## Introduction

Type 2 diabetes is a growing public health burden. Its global prevalence among adults has been estimated at 537 million and is predicted to rise to 643 million by 2030 and 783 million by 2045 [1]. According to the 2023 Malaysian National Health and Morbidity Survey (NHMS), 15.6% of adults aged 18 years and above have been diagnosed with diabetes [2]. The prevalence of diabetes increases with age, affecting one in three Malaysians aged 60 years or over, twice as high as that reported in worldwide statistics (15%) [3, 4].

A fall is defined as “an unexpected event in which the participants come to rest on the ground, floor, or lower level” [5]. Falls are the second leading cause of accidental deaths worldwide. Complications from falls include decreased functional status, serious injuries, increased utilisation of medical services, all of which affect mortality, morbidity, independence and quality of life. Most falls result from the interaction of multiple risk factors that include age, impaired mobility, deconditioning, medical comorbidities, impaired vision, medications, social isolation, fear of falling, foot problems and nutrition [6]. Up to a third of older people fall at least once per year reflecting the increased prevalence of these risk factors and their cumulative effect on risk with increasing age. Diabetes has been identified as a risk factor for falls. A meta-analysis of falls in a diabetic population showed that the incidence of falls in diabetics was significantly greater than in non-diabetics [2]. This risk is further increased in diabetics who are on insulin. Other studies have shown that falls occur more frequently with poor diabetes control and mobility impairment [7].

It is postulated that diabetes directly affects falls risk due to its effect on sensorimotor function (loss of proprioception due to peripheral neuropathy, loss of vision due to retinopathy), neuromuscular function (physical activity, muscular control) and diabetic foot disease [8]. Additionally, autonomic dysfunction can lead to postural hypotension and syncope, and hypoglycaemia due to insulin use can lead to a decreased state of consciousness. The consequences of a fall in diabetics are a cause for concern with diabetics being at higher risk of fractures [9]. In addition, individuals with diabetes who fall, also experience longer hospital stays following hip fracture surgery and have poorer functional outcomes after rehabilitation [10, 11].

The above studies, which have evaluated risk factors for falls in diabetes, have primarily addressed diabetes treatment and complications. Little is known about the role of psychological factors, social factors and health-seeking behaviour in falls risk among individuals with diabetes. Therefore, this study aimed to determine the influence of psychosocial factors and health-seeking behaviour on the risk of falling among individuals aged 55 years and over with type 2 diabetes.

## Methods

This prospective longitudinal study utilised baseline and follow-up data from the Malaysian Elders Longitudinal Research (MELoR). The MELoR study is an interdisciplinary research initiative which examined ageing-related issues among the older communities surrounding Universiti Malaya. Participants were residents aged 55 years and above from the parliamentary constituencies of Petaling Jaya North, Petaling Jaya South, and Pantai Valley in Kuala Lumpur. These participants were selected through simple random sampling stratified by age deciles and the three main ethnicities: Malay, Chinese and Indian. A detailed description of the cohort study can be accessed elsewhere [12]. This study is approved by the University of Malaya Medical Centre Ethics Committee (reference number: 943.6).

### Data collection

Participants were contacted and visited initially at their own homes during initial recruitment. Written informed consent was obtained from all participants at baseline and follow-up. Baseline data was collected in 2013–2015 with computer-assisted home-based interviews and the follow-up in 2019 via telephone interviews by trained researchers. Information on sociodemographic, medical and medication history, falls characteristics and outcomes, physical activities, psychosocial and health-seeking behaviour were collected during the baseline survey. Participants were interviewed in their preferred language of English, Bahasa Malaysia, Chinese or Tamil. Survey questions and responses were recorded directly through the REDCap web-based application (Vanderbilt University, Texas, USA). Data was then stored within a secure university server for a minimum of seven years.

### Case definitions

Diabetes was defined using self-reported physician diagnosis, medication use or glycated haemoglobin (HbA1C) levels ≥6.3% [13]. The primary outcome measure was fall occurrence at first year follow-up, which was considered the occurrence of at least one fall the preceding year. These individuals were deemed fallers in this research. The secondary outcome considered in this study was recurrent falls defined as two or more falls occurring in the year preceding the timepoint of the interview.

### Psychological measures

Anxiety: The 21-item Depression Anxiety and Stress Scale (DASS-21) was used to measure anxiety at baseline. This scale is divided into three subscales which are for depression, stress and anxiety. Each of these subscales included seven items rated with a Likert scale ranging from 0 (did not apply to me at all) to 3 (applied to me very much or most of the time). During follow-up virtual interviews, only seven items in the anxiety subscale were administered [14]. Therefore, the maximum total score for each subscale was 21.

Stress: The DASS-21 scale and the Perceived Stress Scale (PSS-4) [15] were used to measure stress at baseline and follow-up, respectively. PSS-4 is a four-item scale that measure stress based on a Likert-type scale ranging from 0 (never) to 4 (very often). The maximum score for PSS-4 was 16 with higher scores indicating higher stress level.

Depression: The DASS-21 scale and the 15-item Geriatric Depression Scale (GDS-15) were used to measure depression at baseline. Each item is GDS-15 draw a dichotomous response of “Yes” or “No”. This scale consists of 10 positive response items and 5 negative response items were assigned scores of one, with scores of five or more indicating the presence of depression [16, 17].

### Physical activity

The International Physical Activity Questionnaire (IPAQ) was used to measure physical activity (walking, moderate, and vigorous activities) and estimated time spent sitting per week. The IPAQ was scored in total metabolic equivalent (MET) per week as per the author’s recommendations [18]. Then, the total physical activity was reported in three levels: 1) inactive, 2) minimally active and 3) health-enhancing physical activity (HEPA) active.

### Social support

Social support was measured using the six-item Lubben’s Social Network Scale (LSNS-6) [19]. This six-point Likert scale was used to assess the number of relatives and friends the individual had contact with, felt at ease with and could call on for help for the past month. The scores for total of all items as well as friends and family domains calculated. A cut-off score of less than 12 was considered at risk of social isolation, and subscale scores of less than 6 were used to determine family and friends isolation.

### Health utilization

Healthcare utilization was determined through self-reported attendance to private or public hospitals or clinics and traditional or alternative medicine practitioners.

### Data analysis

Data were analyzed using the Statistical Package for Social Sciences (SPSS) version 21 (IBM, United States). Sociodemographic variables, fall occurrences and characteristics among older persons living with diabetes were compared to individuals without diabetes. The risk of falls at follow-up associated with type 2 diabetes was then determined. Continuous variables were expressed in mean and standard deviation, whilst categorical data were expressed in frequency and percentage. Univariate analysis between continuous and categorical data was performed using the independent t-test whilst the chi-square test was used to determine if there was any association between two categorical variables. Multiple logistic regression was then performed to determine the association between falls and risk factors among type 2 diabetes individuals. Variables were selected for multivariate analysis based on clinical judgement and statistical differences (*p* < 0.05) within Table 3. A *p*-value of <0.05 was regarded as statistically significant.

## Results

**Fig. 1:**
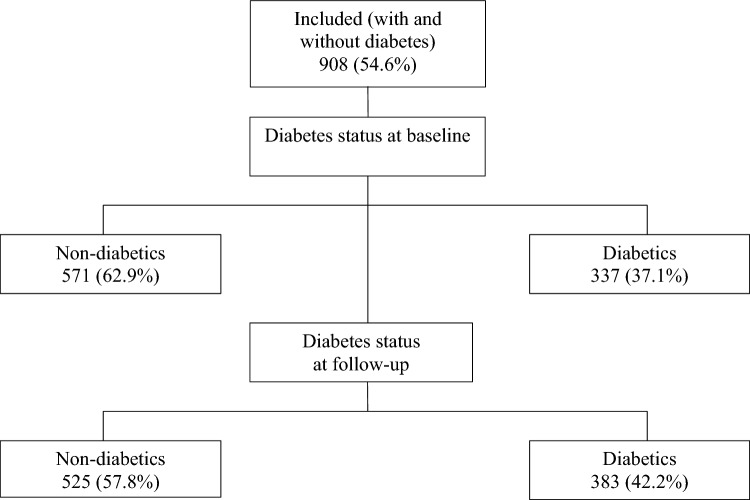
Flow diagram for included participants and diabetes status

Data were available for 908 participants at both baseline and follow-up. This study found that diabetes was present in 37.1% of participants at baseline, which increased to 42.2% at follow-up (Fig. 1).

The demographic and clinical characteristics of social isolation, health utilization and psychological factors of the 908 participants based on their diabetes status at follow-up are shown in Table 1. There were significant differences in age, education level, ethnicity, hypertension, hyperlipidaemia, myocardial infarction, comorbidities, and alcohol consumption among diabetics and non-diabetics at follow-up. It was also found that those with diabetes had significantly higher rates of healthcare utilisation, depression and stress when compared to non-diabetics at follow-up. However, it was found that there were no significant differences in social isolation between diabetic and non-diabetics at follow-up.

**Table 1:** Demographic, clinical characteristics, social isolation, health utilization and psychological factors of participants according to diabetes status at follow-up

Variable	Total n (%) n = 908	Diabetic n (%) n = 383	Non-diabetic n (%) n = 525	*p*-value
Mean age (years) [SD]	68 (7.3)	69 (7.4)	68 (7.2)	0.036*
Gender				
Female	509 (57.4)	199 (53.6)	310 (60.2)	0.052
Education level				
No formal education/Primary	183 (20.6)	97 (26.0)	86 (16.7)	0.002*
Secondary	403 (45.4)	165 (44.2)	238 (46.3)	
Tertiary	301 (33.9)	111 (29.8)	190 (37.0)	
Marital status				
Single/Divorced/Widowed	211 (23.9)	93 (25.2)	118 (23.40)	0.450
Married	671 (76.1)	276 (74.8)	395 (77.0)	
Ethnicity				
Indian	288 (32.6)	162 (43.8)	140 (24.5)	<0.001*
Chinese	382 (43.2)	101 (27.3)	281 (54.7)	
Malay	210 (23.8)	107 (28.9)	103 (20.0)	
*Types of comorbidities				
Hypertension	455 (52.0)	241 (65.3)	214 (42.3)	<0.001*
Hyperlipidaemia	414 (47.5)	202 (55.0)	212 (42.0)	<0.001*
Arthritis (OA/RA)	145 (16.7)	69 (19.0)	76 (15.1)	0.138
Osteoporosis	84 (9.7)	30 (8.3)	54 (10.7)	0.225
Myocardial infarction	50 (5.7)	26 (7.9)	24 (4.4)	0.029*
Cerebrovascular disease	35 (4.0)	19 (5.1)	16 (3.1)	0.133
≥3 comorbidities	129 (14.6)	70 (18.9)	59 (11.5)	0.002*
Currently consuming alcohol	174 (23.2)	56 (18.4)	118 (26.6)	0.009*
Current smoker	48 (5.6)	18 (5.0)	30 (6.0)	0.546
Still working	195 (24.0)	75 (22.5)	120 (24.9)	0.425
Social isolation	206 (23.4)	98 (26.6)	108 (21.1)	0.054
Family isolation	282 (31.9)	121 (32.8)	161 (31.3)	0.644
Friends isolation	265 (30.0)	117 (31.7)	148 (28.7)	0.342
IPAQ category				
Inactive	666 (73.3)	293 (76.5)	373 (71.0)	0.184
Minimally active	184 (20.3)	63 (17.8)	116 (22.1)	
HEPA active	58 (6.4)	22 (5.7)	36 (6.9)	
Exercise regularly (≥3 times/week)	404 (45.7)	158 (42.2)	246 (48.2)	0.077
Healthcare utilization				
Sought medical care in the past 12 months	760 (83.7)	332 (86.7)	428 (81.5)	0.038*
Types of medical facilities utilized				
Public hospital	415 (45.7)	196 (51.2)	219 (41.7)	0.005*
Private hospital	185 (20.4)	81 (21.1)	104 (19.8)	0.621
Public clinic	153 (16.9)	74 (19.3)	79 (15.0)	0.089
Private clinic	247 (27.2)	93 (24.3)	154 (29.3)	0.091
Traditional medical practitioner	23 (2.5)	7 (1.8)	16 (3.0)	0.248
Mental health status				
Depression, anxiety or stress	185 (21.5)	85 (23.5)	100 (20.1)	0.237
Depression	76 (8.7)	41 (11.1)	35 (7.0)	0.031*
Stress	47 (5.4)	27 (7.3)	20 (4.0)	0.030*
Anxiety	150 (17.3)	69 (18.8)	81 (16.2)	0.311

^*^p < 0.05 = statically significant; OA = osteoarthritis; RA = rheumatoid arthritis. IPAQ = international physical activity questionnaire

A total of 22.8% had reported having fall at least once in the past 12 months at baseline, while 25.3% had at least one fall in the past 12 months at five-year follow-up. The risk of falls was higher in diabetics compared to non-diabetics at baseline (OR: 1.484: 95% CI: 1.060–2.077) and follow-up (OR: 1.424; 95% CI: 1.038–1.954). There was a significant difference between diabetics and non-diabetics in recurrent falls at baseline (OR: 2.187, 95% CI: 1.290–3.707) but not at follow-up (OR: 1.453; 95% CI: 0.910–2.319) (Table 2).

**Table 2:** Prevalence of diabetes and falls at baseline and follow-up

	Baseline; n (%) (n = 908)	OR (95% Cl)	*p*-value	Follow-up; n (%) (n = 908)	OR (95% Cl)	*p*-value
Diabetes	337 (37.1)	–	–	383 (42.2)	–	–
Any falls						
Diabetic	77 (22.8)	1.484 (1.060–2.077)	0.021*	97 (25.3)	1.424 (1.038–1.954)	0.029*
Non-diabetic	95 (16.6)					
Recurrent falls						
Diabetic	33 (9.8)	2.187 (1.290–3.707)	0.004*	39 (10.2)	1.453 (0.910–2.319)	0.117
Non-diabetic	27 (4.7)					

Table 3 reported the demographic, clinical characteristics, psychosocial factors, health utilization and prospective falls in diabetics and non-diabetics. The prevalence of falls at follow-up among diabetics was 97 (25.3%) compared to 101 (19.2%) in non-diabetics. It was found female gender, arthritis, alcohol consumption and presence of depression, anxiety or stress in individuals with diabetes significantly increased the risk of falls. Arthritis was the only significant factor associated with falls among non-diabetics.

**Table 3:** Association of prospective falls (at follow-up) with patient demographic, clinical characteristics, social isolation, health utilization and psychological factors

	Diabetic n (%) n = 383			Non-diabetic n (%) n = 525		
	Faller n = 97	Non-faller n = 286	*p*-value	Faller n = 101	Non-faller n = 424	*p*-value
Mean age (years) [SD]	70 (8.2)	69 (7.1)	0.095	68 (7.1)	69 (7.7)	0.197
Gender						
Female	58 (64.4)	141 (50.2)	0.018*	33 (32.7)	172 (41.5)	0.102
Education level						
No formal education/Primary	29 (31.5)	68 (24.2)	0.305	20 (19.8)	66 (16.0)	0.409
Secondary	40 (43.5)	125 (44.5)		49 (48.5)	189 (45.8)	
Tertiary	23 (25.0)	88 (31.3)		32 (31.7)	158 (38.3)	
Marital status						
Single/Divorced/Widowed	29 (32.2)	64 (22.9)	0.078	25 (24.8)	93 (22.6)	0.641
Married	61 (67.8)	215 (77.1)		76 (75.2)	319 (77.4)	
Ethnicity						
Indian	42 (46.7)	120 (42.9)	0.748	21 (20.8)	82 (19.9)	0.114
Malay	26 (28.9)	81 (28.9)		47 (46.5)	234 (56.7)	
Chinese	22 (24.4)	79 (28.2)		33 (32.7)	93 (22.5)	
*Types of comorbidities						
Hypertension	59 (65.6)	182 (65.2)	0.955	40 (40.0)	174 (42.9)	0.604
Hyperlipidaemia	49 (55.1)	153 (55.0)	0.957	40 (40.0)	172 (42.5)	0.654
Arthritis (OA/RA)	25 (28.1)	44 (16.0)	0.011*	24 (24.7)	52 (12.8)	0.003*
Osteoporosis	9 (10.3)	21 (7.6)	0.419	16 (16.0)	38 (9.4)	0.057
Myocardial infarction	7 (7.8)	21 (7.5)	0.931	3 (3.0)	19 (4.6)	0.471
Cerebrovascular disease	8 (8.9)	11 (3.9)	0.064	5 (5.0)	11 (2.7)	0.241
≥3 comorbidities	20 (22.2)	50 (17.9)	0.358	15 (14.9)	44 (10.7)	0.246
Currently consuming alcohol	5 (6.8)	51 (22.0)	0.004*	23 (24.2)	95 (27.2)	0.556
Current smoker	3 (3.5)	15 (5.5)	0.454	5 (5.0)	25 (6.3)	0.623
Still working	15 (19.2)	60 (23.5)	0.426	18 (19.6)	102 (26.2)	0.185
Social isolation	27 (29.7)	71 (25.6)	0.450	27 (26.7)	81 (19.7)	0.118
Family isolation	26 (28.6)	95 (34.2)	0.323	30 (29.7)	131 (31.7)	0.695
Friends isolation	35 (38.5)	82 (29.5)	0.111	33 (32.7)	115 (27.8)	0.330
IPAQ category						
Inactive	74 (76.3)	219 (76.6)	0.406	75 (74.3)	298 (70.3)	0.424
Minimally active	15 (15.5)	53 (18.5)		22 (21.8)	94 (22.2)	
HEPA active	8 (8.2)	14 (4.9)		4 (4.0)	32 (7.5)	
Exercise regularly (≥3 times/ week)	43 (46.7)	115 (40.8)	0.315	47 (48.0)	199 (48.3)	0.951
Healthcare utilization						
Sought medical care in the past 12 months	85 (87.6)	247 (86.4)	0.751	87 (86.1)	341 (80.4)	0.184
Types of medical facilities utilized						
Public hospital	56 (57.7)	140 (49.0)	0.135	49 (48.5)	170 (40.1)	0.123
Private hospital	15 (15.5)	66 (23.1)	0.113	21 (20.8)	83 (19.6)	0.783
Public clinic	19 (19.6)	55 (19.2)	0.939	17 (16.8)	62 (14.6)	0.577
Private clinic	23 (23.7)	70 (24.5)	0.879	27 (26.7)	127 (30.0)	0.523
Traditional medical practitioner	2 (2.1)	5 (1.7)	0.842	4 (4.0)	12 (2.8)	0.553
Mental health status						
Depression, anxiety or stress	32 (35.6)	53 (19.5)	0.002*	24 (25.0)	76 (19.0)	0.184
Depression	15 (16.5)	26 (9.4)	0.062	9 (9.4)	26 (6.4)	0.304
Stress	11 (12.2)	16 (5.8)	0.041*	6 (6.2)	14 (3.4)	0.215
Anxiety	27 (29.7)	42 (15.2)	0.002*	19 (19.8)	62 (15.3)	0.283

### Multivariate analysis

Multivariate logistic regression using the overall population revealed that the relationship between diabetes and any falls at follow-up was no longer significant after adjustment of age and gender differences (OR: 1.327; 95% CI: 0.958–1.838, *p* = 0.089). Table 4 summarizes the logistic regression analysis with falls among diabetics as the dependent variable, and the presence of any depression, anxiety or stress as the independent variable first unadjusted followed by adjustments for potential confounders and mediators. Falls among individuals with diabetes remained significantly associated with the presence of any depression, anxiety or stress after adjustments for age, gender, ethnicity, and education (OR: 1.947; 95% CI: 1.115–3.402) but this relationship was attenuated after additional adjustment for arthritis (OR: 1.763; 95% CI: 0.996–3.122).

**Table 4:** Adjusted odds ratios of occurrence of falls (at follow-up) among diabetes

	Diabetic fallers, OR (95% CI)	*p*-value
Unadjusted	2.280 (1.348–3.856)	0.002
Model 1	2.097 (1.217–3.614)	0.008
Model 2	2.011 (1.159–3.489)	0.013
Model 3	2.001 (1.149–3.484)	0.014
Model 4	1.947 (1.115–3.402)	0.019
Model 5	1.763 (0.996–3.122)	0.052

Model 1: Adjusted for age

Model 2: Model 1 + gender

Model 3: Model 2 + ethnicity

Model 4: Model 3 + education level

Model 5: Model 4 + arthritis

## Discussion

The prevalence of falls among older persons with diabetes was found to have increased from 22.8 to 25.3% at 5-year follow-up. There was a significantly higher risk of falls among diabetics compared to non-diabetics at baseline and follow-up. Meanwhile, there was a significant difference in recurrent falls between diabetics and non-diabetics at baseline but not follow-up. Female gender, arthritis and alcohol consumption, presence of any depression, anxiety or stress were significantly associated with an increased risk of falls among diabetics whilst arthritis was the only significant factor associated with falls among non-diabetics. Falls among diabetes remained significantly associated with the presence of any depression, anxiety or stress after adjustments for age, gender, ethnicity, and education but this relationship was attenuated after additional adjustment for arthritis. These individuals the potentially important role of arthritis in defining falls risk among individuals with diabetes aged 55 years and over.

Previous studies have found that there is an increased risk of falls among older persons with diabetes. A systematic review found that older people with diabetes often experience more falls (25%) when compared to those without diabetes (18.2%) [2]. In Malaysia, similar findings were found in a cross-sectional study conducted in the East Coast of Peninsular Malaysia where the prevalence of falls among older persons with diabetes was 18.8% [20]. Longitudinal studies in China found that the prevalence of falls among older persons living with diabetes was 29.4%, with a significantly higher risk of falls in individuals with diabetes compared to those without diabetes after 4 years of follow-up [21, 22]. The increased risk of falls in individuals with diabetes have been found to be associated with established diabetic complications including peripheral neuropathy, diabetic retinopathy, hypoglycaemia and polypharmacy [23, 24].

Overall, it is observed that Asian populations (13.89%) have a lower incidence rate of falls compared to other ethnic groups like Hispanics (18.54%), Blacks (18.60%) and Caucasians (23.77%) [25]. The potential reasons for the observed ethnic differences may include differences in attitudes toward falls risk and participation in risk-taking behaviours between Asian and Caucasian groups [26]. Lower recurrent falls rates in the Asian group may be due to fear of falling, adult children preventing older adults living with them from undertaking activities seen as dangerous and cultural behaviours such as being more likely to use walking sticks [26].

Previous studies have highlighted the importance of falls risk assessments in individuals with diabetes [27]. Regular screening in diabetes clinics includes peripheral neuropathy and vision screening while gait, balance, and ankle muscle strength assessments may also help minimise the risk of falls and prevent fractures [27, 28]. Frequent medication reviews, careful control of glucose levels to avoid hypoglycaemia and regular blood pressure checks to prevent hypotension are also likely to help reduce falls risk [29]. Arthritis is a common cause of skeletal muscle functioning, leading to postural instability and increased falls risk among older adults [30]. This study suggests that it may also be beneficial to screen for the presence of psychological issues in individuals with diabetes as a fall prevention measure. Psychological status and muscle strength may be modifiable risk factors to prevent arthritis from worsening among older persons [30, 31]. Depression was found to be associated with excessive fear of falling and social isolation due to dietary restrictions [32]. Impaired gait and balance are linked to both depression and fear of falling which is mediated by motor, sensory, and cognitive pathways [32]. The management of depression in fall-prone individuals can also be challenging as antidepressants can cause confusion, unsteady gait, and loss of balance with the undesirable consequence of increasing the risk of falls [33]. Proactive care in older persons with diabetes should also include falls education given the established knowledge of the benefit of reducing falls risk. An effective way to introduce this is to make falls education part of a targeted multicomponent falls prevention exercise programme to improve balance and walking among older persons with diabetes [34]. The World Falls Guidelines recommend that healthcare professionals to ask all older people or their caregivers at least once a year about falls and the frequency of falling [35].

While there may be concerns that falls were determined through retrospective recall within this study leading to recall bias, it was found that fall detection using retrospective recall within the MELoR cohort was comparable to falls calendar recording with more individuals successfully followed-up [36]. The potential benefit of screening and management of arthritis and psychological status in among individuals aged 55 years with diabetes should now be evaluated in future intervention studies, as a potential strategy toward reducing the falls in those living with diabetes. With the available evidence of increased risk of falls in diabetes, such an approach could be useful in reducing healthcare utilization and enhancing quality of life in those living with diabetes.

## Conclusion

Individuals aged 55 years and over with diabetes were at an increased risk of falls at 5-year follow-up when compared to non-diabetics. The psychosocial factors of the presence of depression, anxiety or stress was found to be significant with falls within the individuals with diabetes within this study population. The presence of arthritis accounted for the prospective increased in falls with the presence of depression, anxiety or stress among those with diabetes., Future studies should consider regular screening psychological issues and arthritis among individuals living with diabetes aged 55 years, while effective strategies for the prevention and management of arthritis and psychological problems should now be developed.

## Data Availability

No datasets were generated or analysed during the current study.
